# Improved spatial memory, neurobehavioral outcomes, and neuroprotective effect after progesterone administration in ovariectomized rats with traumatic brain injury: Role of RU486 progesterone receptor antagonist

**DOI:** 10.22038/ijbms.2021.50973.11591

**Published:** 2021-03

**Authors:** Ladan Amirkhosravi, Mohammad Khaksari, Vahid Sheibani, Nader Shahrokhi, Mohammad Navid Ebrahimi, Sedigheh Amiresmaili, Neda Salmani

**Affiliations:** 1 Neuroscience Research and Physiology Research Centers, Kerman University of Medical Sciences, Kerman, Iran; 2 Endocrinology and Metabolism Research Center, Institute of Basic and Clinical Physiology Sciences, Kerman University of Medical Sciences, Kerman, Iran; 3 Neuroscience Research Center, Kerman University of Medical Sciences, Kerman, Iran; 4 Physiology Research Centers, Institute of Basic and Clinical Physiology Sciences, Kerman University of Medical Sciences, Kerman, Iran; 5 Department of Physiology, Bam University of Medical Sciences, Bam, Iran; 6 Department of Psychology, Genetic Institute, Islamic Azad University- Zarand Branch, Kerman, Iran

**Keywords:** Behavioral disorders, Mifepristone, Neuroprotection, Progesterone, Spatial memory, TBI

## Abstract

**Objective(s):**

The contribution of classic progesterone receptors (PR) in interceding the neuroprotective efficacy of progesterone (P4) on the prevention of brain edema and long-time behavioral disturbances was assessed in traumatic brain injury (TBI).

**Materials and Methods:**

Female Wistar rats were ovariectomized and apportioned into 6 groups: sham, TBI, oil, P4, vehicle, and RU486. P4 or oil was injected following TBI. The antagonist of PR (RU486) or DMSO was administered before TBI. The brain edema and destruction of the blood-brain barrier (BBB) were determined. Intracranial pressure (ICP), cerebral perfusion pressure (CPP), and beam walk (BW) task were evaluated previously and at various times post-trauma. Long-time locomotor and cognitive consequences were measured one day before and on days 3, 7, 14, and 21 after the trauma.

**Results:**

RU486 eliminated the inhibitory effects of P4 on brain edema and BBB leakage (*P*<0.05, *P*<0.001, respectively). RU486 inhibited the decremental effect of P4 on ICP as well as the increasing effect of P4 on CPP (*P*<0.001) after TBI. Also, RU486 inhibited the effect of P4 on the increase in traversal time and reduction in vestibulomotor score in the BW task (*P*<0.001). TBI induced motor, cognitive, and anxiety-like disorders, which lasted for 3 weeks after TBI; but, P4 prevented these cognitive and behavioral abnormalities (*P*<0.05), and RU486 opposed this P4 effect (*P*<0.001).

**Conclusion:**

The classic progesterone receptors have neuroprotective effects and prevent long-time behavioral and memory deficiency after brain trauma.

## Introduction

Secondary damage after traumatic brain injury (TBI) initiates an endogenous inflammatory response that yields the rupture of the blood-brain barrier (BBB) and increment of edema in the brain. This is defined clinically as an increased intracranial pressure (ICP), which results in a decline in cerebral perfusion pressure (CPP), brain hypoxia and ischemia (1), and ultimately causes apoptosis (2). Reducing brain edema is a major factor in decreasing mortality after TBI and controlling this edema is a major therapeutic factor in reducing mortality due to TBI.

Behavioral, locomotive, and cognitive activities are disturbed in the short and long terms, depending on the degree of TBI (3). Cognitive deficits such as thinking, memory, and resolving problems, which include mental health or behavioral disorders, are the most prevalent outcomes of brain trauma (4). Neurons of the hippocampus are highly susceptible to traumatic destruction and are amongst the first neurons in the brain to decrease after TBI (5). Hicks *et al*. reported that neuronal decease in that area after TBI results in impaired learning and memory (6). TBI also causes neuropsychiatric problems, such as anxiety disorders, which negatively affect cognitive function and behaviors (7).

Progesterone (P4) has been found to have neuroprotective effects in a variety of clinical and pre-clinical studies (8). This steroid exhibits various neuroprotective properties, including decreasing inflammatory factors (9) and pro-inflammatory cytokines such as transforming growth factor-β (TGF)-β, tumor necrosis factor-α (TNF-α), interleukin-6 (IL-6), and interleukin-1 β (IL-1β) after TBI (10, 11), reducing brain edema (10) by stabilizing BBB (12), decreasing Aquaporin-4 (AQP4) (13), moderating the formation of NO (14) and the damage caused by free radicals (15), decreasing neuronal loss (2), recovering BBB function (16), and preventing necrosis and cellular apoptosis (17). In addition, P4 reduces cognitive defects (17). 

P4 applies its effects to the central nervous system through both classic and non-classic pathways (18). Progesterone receptors (PRs) are revealed in the Hippocampal area of the brain (19). In the mice, the classic progesterone receptors of which have been knocked out, the brain damage and motor impairment increased after ischemia (20). The P4 receptor antagonist, RU486 (mifepristone), is a synthetic steroid derivative that could reduce the neuroprotective effects of P4 (21). Besides, this molecule reduces the neuroprotective effects of progesterone against oxygen-glucose deprivation/re-oxygenation-induced neuronal cell death (21). 

In previous studies, it has been found that P4 has a neuroprotective role in the short-time after TBI (22, 12); accordingly, the present study aimed to evaluate the efficacy of P4 on cognitive and locomotor activities in long term after TBI. Moreover, the role of classical P4 receptors is investigated in interceding the neuroprotective efficacy of this hormone for preventing brain edema, BBB disruption, and long-time behavioral disorders including motor activity, anxiety-like behaviors, and cognitive function.

## Materials and Methods

***Animals***

In this study, the experiments were conducted on the animals in agreement with the Guidelines of Animal Experiments (ethical code: IR.KMU.REC.1396.1540) at Kerman University of Medical Sciences. Adult female Wistar rats were holed in an animal house with suitable ventilation, temperature of 23±2 °C, and 12 hr dark/light cycle. They had easy access to water and food.

***Bilateral removal of ovaries***

After the acclimatization period of two weeks, bilateral ovariectomy was done under ketamine/xylazine (80/10 mg/kg, intraperitoneally (IP)) anesthesia, as explained by Khaksari *et al*. (10). Briefly, ovariectomized (OVX) was performed by using an intra-abdominal approach. All animals underwent ovariectomy two weeks before the experiments (23).

***Study groups and protocol***

**Figure 1:**
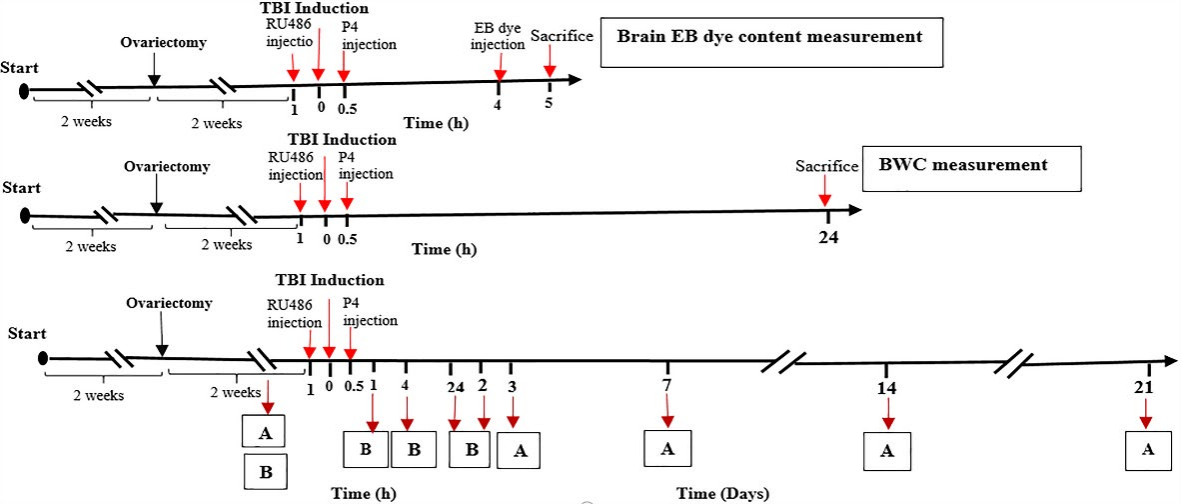
Timeline diagram representing the study design and treatment program. A: MWM, OFT, EPM. B: ICP, CPP, BW

TBI: Traumatic brain injury; EB: Evans blue; BWC: Brain water content; ICP: Intracranial pressure; CPP: Cerebral perfusion pressure; MWM: Morris water maze; OFT: Open field task; EPM: Elevated plus maze; BW: Beam walk task

OVX rats were randomly apportioned into six groups, including 1) Sham: OVX rats attained all essential procedures to create diffuse trauma except dropping weight on their heads. 2) TBI: OVX rats suffering from traumatic brain injury; 3) Oil: OVX rats were administered the same volume of vehicle (sesame oil consumed as estrogen solvent) thirty min following TBI; 4) P4: OVX rats receiving the injection of progesterone (1.7 mg/kg) thirty min following TBI. The dose selected for progesterone was based on our previous study and in the single-dose form (13); 5) Mifepristone (RU486): OVX rats receiving the injection of mifepristone (5 mg/kg) 1 hr before TBI. In all the treatment groups (including RU486), progesterone was injected only once, which was after TBI (24); 6) Vehicle: OVX rats receiving the injection of the same volume of vehicle (dimethyl sulfoxide consumed as mifepristone solvent) 1 hr before TBI. We used 6 rats in each experimental group for assessing BBB integrity, 6 rats for measuring the brain water content, and another 6 rats for registration of ICP, CPP, and evaluating neurobehavioral outcome (Figure 1). It should be noted that the drugs were injected IP. The progesterone and sesame oil were obtained from Aburaihan Pharmaceutical Company (Tehran, Iran). Ru486 was obtained from Sigma.

***Induction of diffuse TBI***

Intubation was done in anesthetized rats (ketamine (50 mg/kg) plus xylazine (5 mg/kg), IP) before TBI. diffuse injury based on Marmarou’s method (25). A 300 g weight was liberated from 2 m above the head of the anesthetized rat while a steel plate, 10 mm in diameter and 3 mm in thickness, was glued to the animals’ skull (between bregma and lambda). After inducing the TBI, all rats were instantly joined to a respiratory pump (TSA animal respiratory compact, Germany). The normal respiration was returned and, then the intratracheal tube was removed. After the recovery, each rat was kept in a solitary cage (26). 

***Assessment of brain edema***

Brain water content was assessed 24 hr after inducing brain trauma (Figure 1). In the anesthetized rats, the brain was instantly removed and weighed (wet tissue weight). The tissue was put in an incubator at 100 °C for 24 hr and reweighed (dry tissue weight). Finally, the percentage of brain water content (BWC) was computed as follows (27):

***Assessment of BBB permeability***

The BBB’s rupture was specified by evaluating the brain’s Evans blue (EB) dye as illustrated formerly (28). Briefly, 4 hr after the trauma (Figure 1), 20 mg/kg EB dye 2% (1 mg/ml) was injected into the jugular vein of the animal under anesthesia with ketamine/xylazine. Then, 1 hr after the injection, saline 0.9% solution was infused into the left ventricle. At the same time, the jugular vein was cut bilaterally to remove the intravenous EB dye. Then, the brain was instantly taken and placed in a shaker in the solution comprising 6 ml of Na_2_SO_4_ %1 + 14 ml acetone for 24 hr. Then supernatant of the centrifuged solution was utilized for evaluating EB dye absorption at 620 nm by a spectrophotometer (Pharmacia Biotech, Germany). Leakage levels of blue dye were assessed for brain tissue (μg / g).

***Histological assay***

The animals were anesthetized with a high dose of ketamine/xylazine twenty-four hr after TBI; then, the brain samples were collected. The brain tissues were cut into 10 μm sections after fixation with 10% buﬀered formaldehyde and staining in paraffin. The sections were dyed with hematoxylin and eosin. Inflammation, edema, and vascular congestion were evaluated in the brain tissues by two pathologists, blinded to the protocol.

*Measurement of intracranial pressure*

ICP was estimated previous to and 1, 4, 24, and 48 hr after the trauma utilizing a pre-prepared monitoring system (Figure 1). The animal was situated in a stereotaxic device and the animal’s head was bent 30 to 40 degrees relative to the horizon. After determining the location of the occipital bone, the end of the polyethylene tube No. 10 connected to the tip of needle No. 20 was gradually pushed into the space of the Cisterna Magna. This system is joined to a pressure transducer and the pressure recording process was performed (22). 

*Determination of cerebral perfusion pressure*

Systolic and diastolic blood pressure was registered by the tail-cuff technique using the NIBP ML125 system (AD Instruments, Australia). In this procedure, the pneumatic pulse sensor and the occlusion cuff were conjoined to the base of the animal’s tail. Mean arterial blood pressure (MABP )was computed, and CPP = MABP - ICP (29). The CPP was registered before and 1, 4, 24, and 48 hr after the trauma (Figure 1).

*Assessment of vestibulomotor function*

Vestibulomotor function and coordination were assessed using the beam walk (BW) task (30), comprising a wooden beam (100 cm long, 2.5 cm wide, and 50 cm high). The beam is connected to a platform on one end, where a black box (as a place for inciting the animal to traverse the beam) opening toward the beam is installed on the platform. Each animal was individually placed on a beam and its ability to get away from bright light and white noise by crossing the beam to arrive at a target box is estimated. Animals were trained prior to TBI or sham surgery (i.e., traverse entire length of the beam under 5 sec). Three trials including d0, d1, and d2 times of post-TBI were executed per animal per day (Figure 1). The execution was assessed by measuring the spent time and the distance traveled from the beam. The animal’s distance traveled was scored on a scale ranging from 0 to 5. Animals favorably arriving at the target box were assigned 5 points, while rats not arriving in the target box were given lesser scores, depending on their final spot on the beam. Spinning on the beam or any limb use on the side of the beam was counted as a fall. The results of each session were tracked utilizing a camera and software (video tracking, BorjeSanat Company, Tehran, Iran). 

*Open field task (OFT)*

One the day before TBI and days 3, 7, 14, and 21 after trauma (Figure 1), each rat was put in the center Plexiglas box (90 ×90×30) (Behboud Tahghigh, Kerman, Iran). The instrument was partitioned by lines into 16 equal squares. All experiments were done in a dimly lightened testing room. Each rat was solely located in the central zone and permitted 5 min of free discovery. Total distance moved (m), the average speed (cm/s), and distance moved in the center zone arena (m) of the OFT were then assessed (31).

*Elevated plus maze (EPM) task*

One the day before TBI and days 3, 7, 14, and 21 after trauma, each rat was put in the center of an elevated 4-arm maze with two open arms and two enclosed arms (50×10 cm) (32) (Figure 1). The tool made of wood and closed arms was surrounded by 40-cm heigh walls. The four arms were joined by a central square platform (10×10 cm). The height of the maze from the floor was 50 cm. All examinations were performed in a testing room lit by a 60-W light bulb located above the center of the EPM. Each rat was put in the center of the maze and was able to move freely between the arms for 5 min. A camera was mounted on the top and center of the maze to transfer rats’ movements to the computer and video tracking software. Anxiety-like behavior was determined via the percentage of time spent in the open arm, termed %OAT (33).

*Morris water maze (MWM)*

MWM is a circular dark pool with a diameter of 160 cm and a height of 60 cm, which is filled up with water at the temperature of 25±2 ºC to the height of 35 cm. A platform with a diameter of 10 cm was placed approximately 2 cm below the water level and 1.5 cm from the edge of the pond. A camera was attached to the top of the maze and was connected to a computer having the video tracking software. The device was apportioned into 4 quarters and the animals were put in one of the four equal quarters, randomly. The platform was placed in the center of the southwest quarter. On the walls of the room, special cues with various geometric images were fixed. The learning phase included three continuous days and each day comprised four continuous trials. For each training trial, the animal slowly entered the water at the beginning point. If the animal did not discover the platform in 60 sec, it was gently directed to the platform and permitted to stay on the platform for 20 sec. The time elapsed to find the platform was recorded during the testing trials by the software and the average of 4 trials per training day was computed. Spatial memory was managed by deleting the platform. During 60 sec, the time spent in the target quarter and the number of entries to the target quarter were recorded (34). Spatial learning was evaluated 4 days before the trauma and spatial memory retention was assessed one day before TBI and on days 3, 7, and 21 after injury (Figure 1).

*Data analysis*

Results were represented as mean±SEM. The integrity of BBB and brain edema were analyzed using one-way ANOVA, and Tukey’s test was utilized for the post-hoc analysis. Repeated measures ANOVA was done to compare the mean variable (ICP, CPP, BW, OF, EPM, and MWM training) between groups at different times and by using the Bonferroni *post-hoc *test. All the discrepancies were presumed significant at *P*-value lesser than 0.05.

## Results

***Brain edema: BWC***

Brain edema displayed significant differences in different groups (F_5, 30_=17.28, *P*<0.001). TBI increased the amount of BWC compared with the sham group (*P*<0.001). P4 inhibited the brain edema, so this indicator’s level in the P4 group was lower than that in the oil group (*P*<0.05). Mifepristone inhibited the effects of P4 on this indicator comparison with the VEH + P4 group (*P*<0.05). 

**Figure 2:**
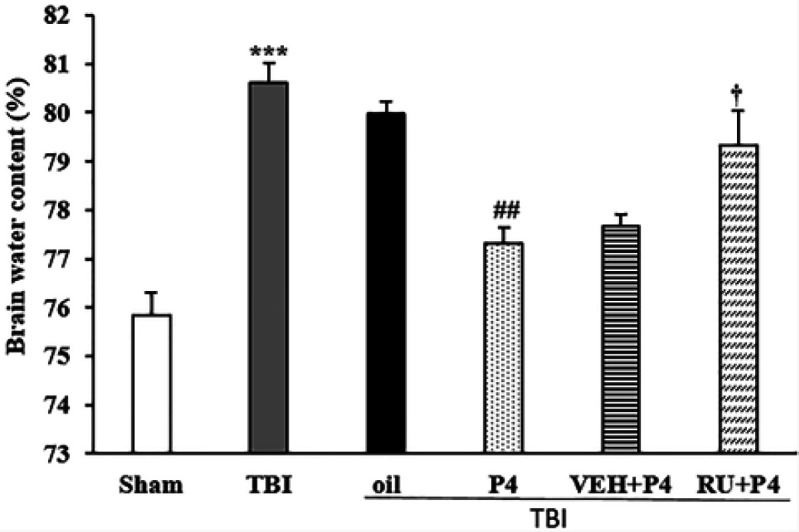
Effects of progesterone and progesterone receptor antagonist on brain water content (%) (n=6 rats per group). Every bar represents mean±SEM.^ ***^ *P*<0.001 compared with Sham group; ^##^ *P*<0.05 compared with oil group; ^†^ *P*<0.05 compared with VEH + P4 group

VEH: Vehicle; P4: Progesterone; RU + P4: RU486 + Progesterone; TBI: Traumatic brain injury

The EB content of the brain displayed significant differences in different groups (F_5, 30_=354.122, *P*<0.001). The EB dye level increased in the TBI group compared with the sham group (*P*<0.001). There was a significant decrease in the P4 group compared with the oil group (*P*<0.001). RU486 eliminated the effects of P4 (*P*<0.001) (Figure 2). 

***Permeability of the BBB: brain EB content***

**Figure 3:**
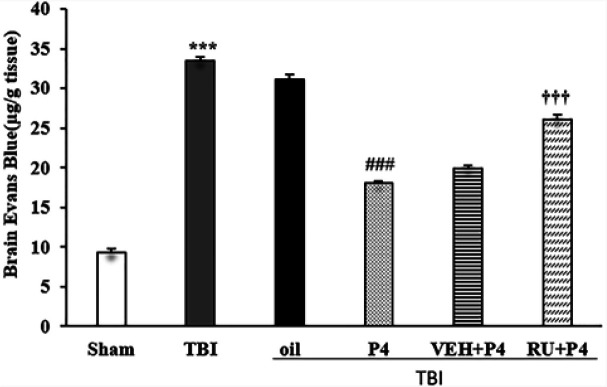
Effects of progesterone and progesterone receptor antagonist on brain EB dye content (n=6 rats per group). Every bar represents mean±SEM.^ ***^ *P*<0.001 compared with Sham group; ^###^ *P*<0.001 compared with oil; ^†††^ *P*<0.001 compared with VEH + P4

TBI: Traumatic brain injury; VEH: Vehicle; P4: Progesterone; RU+P4: RU 486 + Progesterone

The EB content of the brain displayed significant differences in different groups (F_5, 30_ = 354.122, *P*<0.001). The EB dye level increased in the TBI group compared with the sham group (*P*<0.001). There was a significant decrease in the P4 group than the oil group (*P*<0.001). RU486 eliminated the effects of P4 (*P*<0.001) (Figure 3). 

***Histological findings***

**Figure 4:**
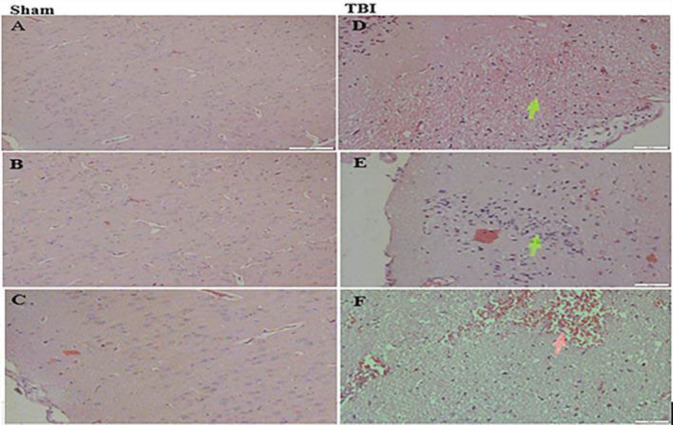
Representative photomicrographs of brain stained by Hematoxylin-Eosin. Sections were obtained 24 hr after TBI or sham surgery. (Magnification 200X). Left side (A, B, and C): sham group, right side: TBI group

Evaluation of the brain sections showed edema, congestion, and inflammation in the TBI group compared with the sham group (Figures 4A, B, and C). The brain vessels were swollen with blood after trauma. Leukocytes, lymphocytes, and plasma cells were infiltrated, and also brain edema (formation of extracellular blanks and separated parenchymal cells) was evident in the TBI group (Figures 4D, E, and F). The sham group represented no aforesaid pathological consequences.

***ICP changes following TBI***

**Figure 5:**
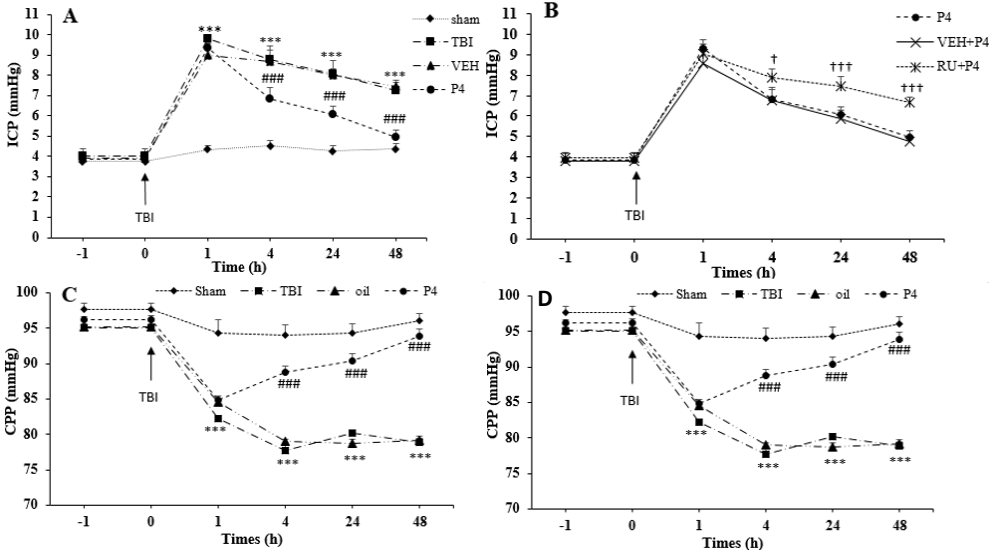
The effect of progesterone and progesterone receptor antagonist on intracranial pressure and cerebral perfusion pressure before and after the brain trauma (n=6 rats per group). Every bar represents mean±SEM. A. ^***^ *P*<0.001 at 1, 4, 24 and 48 hr compared with sham group; ^###^ *P*<0.001 at 4, 24, and 48 hr compared with oil group. B. ^†^* P*<0.05 at 4 hr, and ^†††^ *P*<0.001 at 24, and 48 hr compared with VEH + P4 group. C. ^***^P<0.001 at 1, 4, 24, and 48 hr compared with sham group; ^###^ *P*<0.001 at 4, 24, and 48 hr compared with oil group; D. ^†† ^*P*<0. 01 at 24, and ^†††^ *P*<0.001 48 hr compared with VEH + P4 group

VEH: Vehicle; P4: Progesterone; RU + P4: RU486 + progesterone; TBI: Traumatic brain injury

Repeated measures ANOVA on ICP changes demonstrated the significant effect of time (F_4, 27 _=330.52, *P*<0.001) and groups (F_5,__30_=79.39, *P*<0.001) as well as the interaction among two factors (F_20,__120_= 5.51, *P*<0.001). The amount of ICP was not significant between the groups before trauma; however, TBI increased the amount of ICP at 1 hr, 4 hr, 24 hr, and 48 hr (*P*<0.001 for all times) after trauma compared with the sham group (Figure 5A). P4 prevented the elevation of ICP at 4 hr, 24 hr, and 48 hr (*P*<0.001 for all times) compared with the oil group. RU486 reversed the P4 effects on the reduction of ICP at 4 hr, 24 hr, and 48 hr (*P*<0.05, *P*<0.001, *P*<0.001, respectively) compared with the VEH +P4 group (Figure 5B).

***CPP changes following TBI***

Repeated measures ANOVA on the CPP changes displayed the significant effect of time (F_4__, 120_=221.54, *P*<0.001) and groups (F_5, __30_=122.36, *P*<0.001), also, the interaction among the two factors (F_20,__120_=22.77, *P*<0.0001). Level of CPP decreased at different times of 1 hr, 4 hr, 24 hr, and 48 hr (*P*<0.001 for all times) after trauma compared with the sham group. P4 increased CPP at 4 hr, 24 hr, and 48 hr (*P*<0.001 for all times) compared with the oil group (Figure 5C). This increase was reversed by the injection of RU486 at both times of 24 and 48 hr (*P*<0.001, *P*<0.01, respectively) (Figure 5D).

***Vestibulomotor functions: BW (traversal time and distance traveled)***

**Figure 6:**
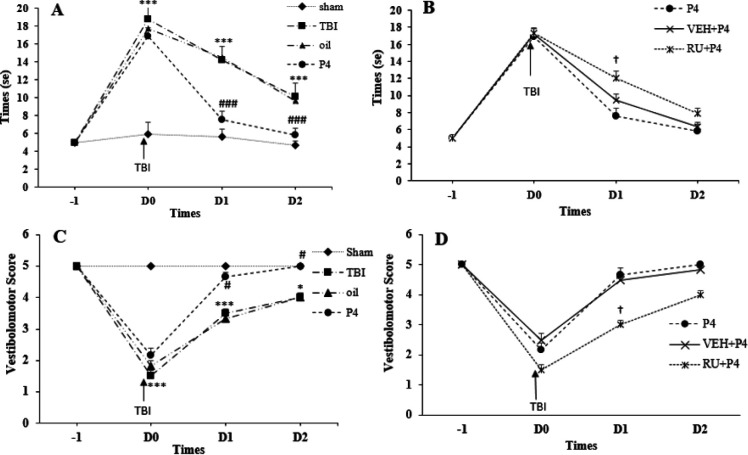
The Effect of progesterone and progesterone receptor antagonist on the time spent and the distance traveled on the elevated narrow path prior to trauma, on day of trauma (d0), first (d1), and second days (d2) after the brain trauma (n = 6 rats per group). Every bar represents the mean±SEM. A.^ ***^P < 0.001 compared with sham group on d0, d1, d2 times. ^###^ *P*<0.001 compared with oil group on d1, d2 times. B. ^†^ *P*<0.001 compared with VEH+P4 group on d1 time. C. ^***^ *P*<0.001 compared with Sham group on d0 and d1 times. ^* ^*P*<0.05 compared with Sham group on d2 time. ^#^ *P*<0.05 compared with oil group on d1, d2 times. D. ^†^ *P*<0.05 compared with VEH + P4 group on d1 time

VEH: Vehicle; P4: Progesterone; RU + P4: RU486 + progesterone; TBI: Traumatic brain injury

Repeated measures ANOVA on traversal time indicated the significant effect of day (F_3, 90_=724.76, *P*<0.001) and groups (F_1, 30_=61.449, *P*<0.001) also, the interaction between day and groups (F_15, 90_=28.11, *P*<0.0001). As indicated in Figure 6A, before the trauma, all the rats arrived at the target box in roughly 5 sec. The traversal time was increased in the TBI group at d0, d1, and d2 (*P*<0.001 for all times) after trauma compared with the sham group. P4 reduced the traversal time on d1 and d2 (*P*< 0.001 for all times) compared with the oil group. RU486 increased the traversal time only on d1 (*P*<0.001) compared with the VEH + P4 group (Figure 6B).

Recorded scores before the trauma in all the groups showed no significant difference, and all the animals with the maximum score of 5 traveled the entire length of the path. In the TBI and oil groups, this index decreased on d0 and d1 (*P*<0.001 for all times) compared with the sham group (Figure 6C). P4 increased the recorded score on d1 and d2 (*P*<0.05 for all times). RU486 reduced the recorded score only on d1 (*P*<0.05) compared with the VEH + P4 group (Figure 6D).

***Locomotor activity***

**Figure 7:**
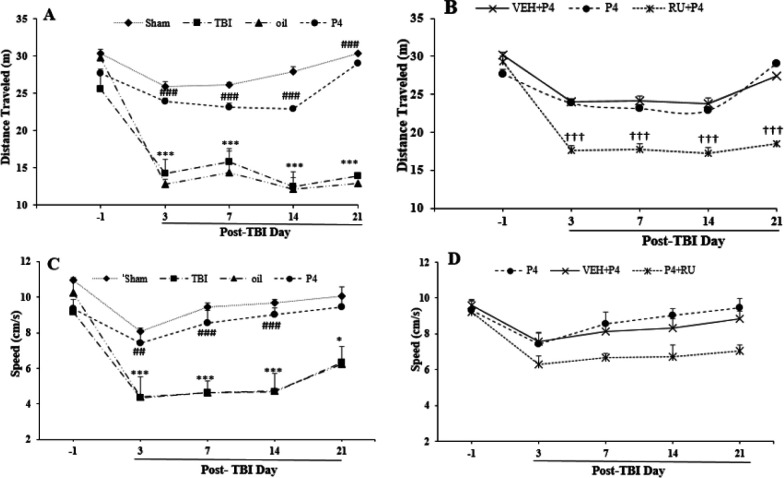
The effect of progesterone and progesterone receptor antagonist on motor behavior (distance traveled, and travel speed) in open field task before and after the brain trauma (n=6 rats per group). Every bar represents the mean±SEM. A. ^***^ *P*<0.001 compared with sham group; ^###^ *P*<0.001 compared with oil group; B. ^†††^ *P*<0.001 compared with VEH+P4 group. C. ^***^ *P*<0.001 and ^*^ *P*<0.05 compared with sham group, ^###^ *P*<0.001 compared with oil group; D. progesterone receptor antagonist did not alter the effect of progesterone on this indicator

VEH: Vehicle; P4: Progesterone; RU + P4: RU486 + Progesterone; TBI: Traumatic brain injury

The analysis of distance traveled on the day before TBI and days 3, 7, 14, and 21 after TBI revealed the significant effect of day (F_4, 27 =_45.6, *P*<0.001) and group (F_5, 30_=59.540, *P*<0.001) as well as the interaction between the two factors (F_20, 120_=3.76, *P*<0.001). The distance traveled in the TBI group decreased on days 3, 7, 14, and 21 (*P*<0.001 for all times) compared with the sham group. In the P4 therapy goup, the distance traveled was increased on days 3, 7, 14, and 21 (*P*<0.001 for all times) compared with the oil group (Figure 7A). RU486 prevented the incremental effects of P4 on 3 (*P*< 0.001), 7 (*P*<0.001), 14 (*P*<0.001), and 21 (*P*<0.001) days (Figure 7B).

The analysis of travel speed during the 5 min test on the days before the trauma and days 3, 7, 14, and 21 after TBI showed the significant effect of day (F_4, 120_=30.59, *P*<0.001) and group (F_5, 30_=37.84, *P*<0.001) as well as the interaction between the two factors (F_20, 120_=2.14, *P*<0.01). As illustrated in Figure 7C, while no difference was found between the groups before the trauma, this index level decreased in the TBI group in comparison with the sham group at days 3, 7, 14 (*P*<0.001), and 21 (*P*<0.05). In the P4 group, the travel speed at days 3 (*P*<0.01), 7, and 14 (*P*<0.001) increased significantly in comparison with the oil group, whereas RU486 could not change the P4 effect (Figure 7D).

***Anxiety-like behavior***

**Figure 8:**
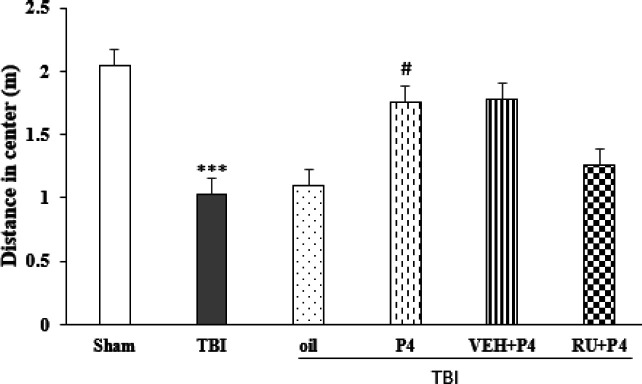
Effect of progesterone and progesterone receptor antagonist on distance moved in the open field center (n=6 per group). Every bar represents the mean±SEM. ^***^P<0.001 compared with sham group. # *P*<0.05 compared with oil group

VEH: Vehicle; P4: Progesterone; RU + P4: RU486+ Progesterone; TBI: Traumatic brain injury

**Figure 9:**
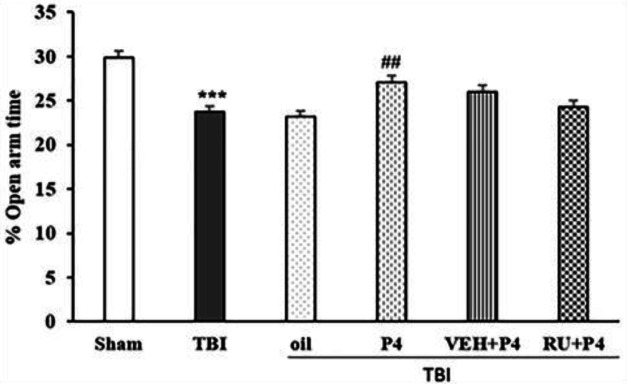
Effect of progesterone and progesterone receptor antagonist on the percentage of open arm time in EPM test (n=6 rats per group). Every bar represents the mean±SEM. *** *P*<0.001 compared with sham group.^ ##^ *P*<0.01 compared with oil group

VEH: Vehicle; P4: Progesterone; RU+P4: RU486+ Progesterone; TBI: Traumatic Brain injury

Repeated measures ANOVA of the distance traveled in the center zone on days before the trauma and days 3, 7, 14, and 21 after TBI showed the interaction between day and group was not significant (F_20, 120_=1.43, *P*=0.11) but effect of the day (F_4, 120_=42.07, *P*<0.001) and group (F5_1,30_=11.85, *P*<0.001) was significant. The distance traveled in the center zone in the TBI group was reduced compared with the sham group (*P*<0.001). In the P4 group, this index was increased compared with the oil group (*P*<0.05). Though, RU486 could not change the effect of P4 on this index (Figure 8). In the other index, repeated-measures ANOVA of %OAT on the day before TBI and days 3, 7, 14, and 21 after TBI showed the interaction between day and group was not significant (F_20, 120_=0.88, *P*=0.25) but effect of the day (F_4,120_=7.91, *P*<0.001) and groups (F_5, 30_=11.55, *P*<0.001) was significant. Trauma reduced %OAT more than in the sham group (*P*<0.001). P4 increased this index more than in the oil group (*P*<0.01); similar to the above index, Ru486 could not inhibit the effect of P4 (Figure 9).

***Spatial memory (MWM test)***

**Figure 10:**
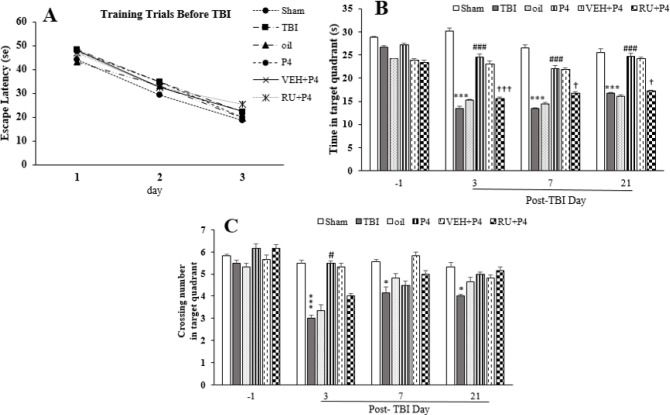
Effect of progesterone and progesterone receptor antagonist on spatial memory in the MWM task. Every bar represents mean±SEM (n=6 rats per group). A. escape latency in the learning phase. Animals in different groups had equivalent learning abilities before the trauma. B. Spatial memory retention was evaluated by measurements of time in target quadrant in different groups before and after the brain trauma; *** *P*< 0.001 compared with sham group; ^###^ *P*<0.001 compared with oil and TBI groups; ^†††^ *P*<0.001, ^†^*P*<0.05 compared with VEH+P4 group; and C. Crossing count of target quadrant; ^***^ *P*< 0.01, ^*^ *P*<0.05 compared with sham group; ^#^ *P*<0.05 compared with oil and TBI groups

VEH: Vehicle; p4: Progesterone; RU + P4: RU486 + progesterone; TBI: Traumatic brain injury

Spatial learning was assessed using the reduction in distance and time taken to find the submerged platform at the three MWM phase blocks. There was no difference between the groups on the days before TBI (F_5, 30_=1.32, *P*=0.27) (Figure 10A). The data for the time spent in the target area are depicted in Figure 10B. Repeated measures ANOVA on the time spent in the target quadrant on days 3, 7, and 21 after the trauma revealed the significant effect of day (F_3, 90_=33.64, *P*<0.001) and group (F_5,30_=90.80, *P*<0.001), as well as the interaction between the day and group (F_15, 90_= 5.06, *P*<0.05). TBI decreased this parameter compared with the sham group on days 3, 7, and 21 (*P*<0.001 for all days) after injury. P4 increased this time at days 3, 7, and 21 (*P*<0.001) compared with the oil group. Use of RU486 eliminated the P4 therapeutic effect on the time spent at days 3, 7, and 21 (*P*<0.001, *P*<0.05, *P*<0.01, respectively) (Figure 10B). Also, this index decreased on the same days in the RU486 group compared with the pre-trauma state.

The data for the crossing number of the target zone is demonstrated in Figure 10C. Analysis of the crossing count of the target zone in the TBI group on days 3, 7, and 21 after the trauma revealed the significant effect of day (F_3, 90_=10.43, *P*<0.001) and group (F_5, 30_=8.71, *P*<0.001), also the interaction between both factors (F_15, 90_=2, *P*<0.05). The crossing number of the target quadrant decreased compared with the sham group at days 3 and 7 (*P*<0.05, *P*<0.01) after trauma. The crossing count of the target zone in the P4 group increased only on day 3 (*P*<0.05) in comparison with the oil group. Meanwhile, RU486 did not affect the number of crossings caused by P4 (Figure 10C).

D: Edema; E: Inﬂammation; and F: Congestion; TBI: Traumatic brain injury

## Discussion

Since TBI induces behavioral and cognitive deficit (35), in the present study, the efficacies of P4 on behavioral and cognitive parameters after TBI were investigated in the presence of classic progesterone receptor antagonist. The principal findings of this study included: 1) Reduction of the brain edema caused by P4 was inhibited by RU486; 2) P4 prevented the disruption of BBB after TBI and this effect, like those of the brain edema, was reversed by RU486; 3) RU486 inhibited the reducing effect of P4 on ICP and its increasing effect on CPP; 4) P4 improved the vestibulomotor function and the receptor antagonist reversed this effect in some cases; 5) The reduced distance traveled induced by trauma was increased by P4 and reduced by RU486; and 6) P4 prevented spatial memory deficits and the use of receptor antagonists opposed this effect.

Destruction of BBB caused brain edema and then an increase in ICP, eventually leading to morbidity and fatality after injury (36). A rise in ICP squeezes brain tissue and reduces CPP and brain blood flow (37). Our results showed that post-TBI administration of P4 in OVX rats applies salutatory effects, perhaps by repressing the brain edema and reducing BBB infiltration. P4 decreases brain edema and cortical contusion after injury (38, 39). Some possible mechanisms, through which P4 controls brain edema, are as follows: reducing the expression of IL-6 and AQP-4 (11, 13), scavenging free radicals and reducing lipid peroxidation (15), decreasing ICP (39), increasing TGF-β, decreasing IL-1β (10), and inhibiting expression of MM-2 and 9 (matrix metalloproteinase 2, 9) (40). 

This study also indicated that classic receptors antagonist inhibited these lowering effects of P4 on brain edema and BBB infiltration. Consistent with the present study, RU486 reduced HI (hypoxic-ischemic)-induced rise in the right hemisphere water content (41). The beneficial effects of P4 on ischemia-induced neuronal death are sensitive to mifepristone (42). Given that RU486 is antagonistic to the anti-edema effect of P4, other than traumatic injuries, it seems that the anti-edema effects observed in this study were in relation to the classic progesterone receptor. In addition, RU486 abolished the anti-edema effect of P4 in meningitis cerebral edema (43). 

Increasing ICP is a determining factor in TBI patients; therefore, in this study, it was shown that ICP increases after trauma, while CPP decreases. Our findings revealed that P4 prevents incremental ICP and decremental CPP, and RU486 inhibits this effect. An increase in ICP and edema after the trauma (22) and also the importance of ICP measurement in TBI along with the association between increased ICP with mortality in TBI and secondary cell death (44) have been reported previously. Furthermore, an association between reduction in brain edema and ICP has been found. Therefore, the primary control of the brain edema accomplishes an essential role in the secondary recovery process (10). The reasons for increasing ICP after TBI are brain edema (45), ischemia or hypoxia (46), and changes in the expression of AQP-4 (47). In contrast to our results, a study showed no increase in ICP after trauma (48). Another parameter that is effective in brain trauma management is CPP, which plays a substantial role in sustaining sufficient cerebral perfusion (44). Therefore, in our study, in addition to ICP, CPP was also measured and it was found that CPP decreased after TBI and increased by progesterone consumption. The mechanisms for decreasing ICP and increasing CPP after TBI by P4 were similar to the mechanisms that could decrease the brain edema. The possible mechanisms, through which P4 decreases CPP after TBI, are via inhibiting the production of free radicals (49), modifying production of NO (50), and diminishing inflammatory cytokines production (51). On the other hand, it has been reported that elevated CPP could not improve post-traumatic outcomes (52). Mifepristone reversed progesterone’s effects on lowering ICP and increasing CPP, which may be partially mediated by classical progesterone receptors. 

TBI causes neuropsychiatric problems such as anxiety disorder that negatively affects cognitive and behavioral functions (7). In another section of our study, it was shown anxiety-like behavior increased EPM and OFT after TBI. Consistent with this study, anxiety-like behavior increased in EPM and OFT after TBI (53). Few studies were inconsistent with this study. Washington *et al*. reported that TBI with different intensities reduced anxiety (54). Another study. did not show any difference in the anxiety-like behavior after TBI (55). These differences may be related to the study type (animal or human), animal sex, time and dose of the drug used, and model and severity of brain injuries. The mechanisms that increase the level of anxiety after the trauma include involvement of the monoaminergic system (53), increased level of stress-related steroids (corticosterone), increased expression of glucocorticoid receptors (56), alterations in the HPA axis (57), and impairment of dopaminergic centers (58). In agreement with the useful role of P4 in improving anxiety after TBI in the present work, the following points can be considered: the elevation of corticosterone level after cerebral ischemia in males is more than in females (59), female rats more spent time in the open arm than male rats (60), and anxiolytic activity in humans and animals (61). The following mechanism(s) have been suggested for P4 effects in terms of reducing anxiety-like behavior: reducing glucocorticoid levels in the brain (62) and increasing the IGF-1 levels (7).

In another part of the study, the results showed that P4 prevented memory impairments induced by TBI in rats by incrementing the time spent in the target zone and also through the number of entries into this zone. But these improving effects of P4 were inhibited by RU486. Traumatic injury has been reported to cause memory deficits, particularly in spatial memory (63). TBI induced cognitive impairment by damaging the hippocampal neurons (6). Besides, stroke and cerebral ischemia can lead to the loss of spatial learning and memory (64, 65). These findings regarding the beneficial effect of P4 on improvement of memory are consistent with improved learning and memory after ischemic injury (66), less spatial memory impairment a long time after TBI (8), reduced spent time to find the platform (67), and improved cognitive function in female mice compared with the male ones after TBI (68) by P4. Moreover, the performance of the two tasks (object placement and novel object recognition) was impaired after inhibiting the activity of the P4 receptor (67). 

The possible mechanism(s), through which TBI leads to learning and memory impairment, include: increased glial fibrillary acidic protein (GFAP) (69), apoptosis (70), increase in Nogo-A, Ng-R, and Rho-A in CA1 neurons (66), increased expression of TNF-α and nuclear factor ƙappa B (NF-ƙB) in the hippocampus (71), apoptosis in the parietal and frontal cortex (72), increased oxidative stress and inflammation (8), and increase in IL-6 brain levels (11). Since motor activity decreased, it is also possible that the decrease in post-traumatic learning and memory may be due to a decrease in motor activity.

The following mechanism(s) have been proposed for the protective effects of P4 in preventing memory deficits: decreasing cell death in the CA1 region (70), increasing expression of brain-derived neurotrophic factor (BDNF) (73), reducing Nogo (A) Ng-R, and Rho-A in the hippocampus (66), decreasing the level of IL-6 and cyclooxygenase 2 (COX2) in the brain (74), activating ERK signaling pathway (75), decreasing oxidative stress and inflammation (8), inhibiting the signaling flow induced by nuclear factor ƙappa B (NF-ƙB) and TNF-α (76), and decreasing GFAP (77).

In agreement with the present study about the role of classic progesterone receptor, the beneficial effect of P4 on motor coordination, induced neurological deficits and infarct volume (20), and increased brain damage and motor impairment after ischemia with the knockout of the classic P4 receptors (78), inhibiting the effects of P4 on neuroinflammation by mifepristone (79), function impairment in object recognition by mifepristone (67), deficits in spatial memory by administration of RU486 (75), neural viability, and expression of AQP-4 by classic progesterone receptors (20) have been reported. Furthermore, PR was characterized to be the mediator of the effect of P4 on BDNF expression, since this effect was inhibited by RU486 (80). PR mediated P4’s effects on Src/ERK1/2 or PI3K/ Akt pathways to control gene transcription and this effect was blocked by RU486 (81). Although in contrast, it has been reported that RU486 did not alter the ability of progesterone to attenuate the calcium signal (potential neuroprotective mechanism), it in fact reveals a PR-independent effect (82). 

## Conclusion

Overall, the present study showed that classic P4 receptors had a mediating P4 effect after TBI. Also, the neural PR was a critical player for the mediation of the beneficial effects of P4 following the brain injury. The inhibition of these receptors after TBI by RU486 eliminated the neuroprotective effects of P4 including edema prevention, decreasing the BBB permeability, decreasing ICP, increasing CPP, enhancing vestibulomotor function, and improving locomotor activity and spatial memory in the long term. Since the anti-progestin activity of RU486 depends on the presence of other steroid hormones, the estrous cycle, and animal’s gender, these cases should be taken into account while using the antagonist. These data indicated that synthetic progestin selectively targeting neural PR may display therapeutic potential after brain trauma. In future research, the non-genomic pathway and intracellular signaling of progesterone receptors could be surveyed.
